# Barriers and facilitators to the use of e-health by older adults: a scoping review

**DOI:** 10.1186/s12889-021-11623-w

**Published:** 2021-08-17

**Authors:** Jessica Wilson, Milena Heinsch, David Betts, Debbie Booth, Frances Kay-Lambkin

**Affiliations:** 1 Centre for Brain and Mental Health Research, University of Newcastle, Callaghan, NSW 2308 Australia; 2 School of Humanities and Social Science, University of Newcastle, Callaghan, NSW 2308 Australia; 3 University Library, University of Newcastle, Callaghan, NSW 2308 Australia

**Keywords:** Mobile health, E-mental health, Acceptance, Engagement, Multimorbidity

## Abstract

**Background:**

Limited attention has been paid to how and why older adults choose to engage with technology-facilitated health care (e-health), and the factors that impact on this. This scoping review sought to address this gap.

**Methods:**

Databases were searched for papers reporting on the use of e-health services by older adults, defined as being aged 60 years or older, with specific reference to barriers and facilitators to e-health use.

**Result:**

14 papers were included and synthesised into five thematic categories and related subthemes. Results are discussed with reference to the Unified Theory of Acceptance and Use of Technology2. The most prevalent barriers to e-health engagement were a lack of self-efficacy, knowledge, support, functionality, and information provision about the benefits of e-health for older adults. Key facilitators were active engagement of the target end users in the design and delivery of e-health programs, support for overcoming concerns privacy and enhancing self-efficacy in the use of technology, and integration of e-health programs across health services to accommodate the multi-morbidity with which older adults typically present.

**Conclusion:**

E-health offers a potential solution to overcome the barriers faced by older adults to access timely, effective, and acceptable health care for physical and mental health. However, unless the barriers and facilitators identified in this review are addressed, this potential will not be realised.

**Supplementary Information:**

The online version contains supplementary material available at 10.1186/s12889-021-11623-w.

## Introduction

In recent years, rapid population ageing has become a worldwide phenomenon. In 2018, older people outnumbered children for the first time in history. By 2050, they are expected to make up 22% of the global population [1]. Commensurate with this growth is the need to ensure proper planning and delivery of health services and supports to facilitate full and happy lives across the age spectrum.

The wellbeing of older adults is diverse. While some lead physically active lives free of major health concerns, population ageing has also coincided with a sharp increase in non-communicable diseases (e.g., diabetes, cancer, and heart disease) [2], and in some older populations, the co-occurrence of multiple chronic conditions is as high as 77% [3]. Age-related factors, such as changes in social roles and familial relationships, retirement, and deteriorating physical health are also associated with increased mental health challenges [4, 5]. Crucially, 15% of older adults experience a mental health disorder [6], and a further 15% experience clinically significant depressive symptoms [7]. This makes the promotion and maintenance of mental health an equally important consideration alongside physical health for older adults. Facilitating access to health and mental health services and supports for older people is, thus, a global imperative.

Currently, health systems are not well aligned with the complex needs of older adults [8]; there is a tendency to focus on individual diagnoses rather than on treatment of the whole person [9–11]. Widespread endorsement of this ‘single disease framework’ by current health systems has arguably hindered the provision of integrated, ‘patient-centred care’ for older adults [11]. Consequently, and despite growing health and medical advances, the rate of mild-to-moderate disability of older adults has remained stable over the past three decades [6, 12], resulting in increased health service utilization [3, 11, 13–15]. At the same time, older adults often face unique challenges to accessing health services, including limited income or insurance, reduced mobility or disability, rural or remote location, and negative self-perceptions of ageing (associated with lower health-related quality of life) [16, 17].

e-Health (defined as any health service, platform, tool, or intervention delivered electronically) [18] has substantial potential to improve access to, as well as support the provision of efficient and effective care for older adults [19, 20]. Research shows that adoption of information and communication technology by older adults is increasing [21], and is perceived to be positive and essential to their everyday lives [22]. This creates significant potential to better support the health care needs of older aged adults within the current limitations of our health service systems. To date, two systematic reviews have explored the benefits of e-health for older adults, finding clinically significant improvements in health behaviors (increased physical activity and healthy eating) as well as psychological and health outcomes (memory and blood pressure) [23, 24] associated with the use of these technologies.

Despite the availability and potential benefits of e-health for older adults [25] barriers to uptake and use remain [23, 26]. Limited attention has been paid to how and why older adults choose to engage with e-health services, and the factors that impact on this. We sought to address this gap by reviewing the existing literature on barriers and facilitators to the use of e-health by older adults, with a view to informing the development and implementation of a targeted e-health intervention for older adults. The results of this review are discussed with reference to the key constructs of the Unified Theory of Acceptance and Use of Technology2 [27].

## Methods

This review follows the Preferred Reporting Items for Systematic Review and Meta-Analyses, Scoping Review extension (PRISMA-ScR) guidelines [28], and uses a scoping review methodology outlined by Arksey and O’Malley [29], and Levac et al. [30]. The choice to conduct a scoping review rather than a systematic review was informed by Munn et al. [31], who explains that systematic reviews focus on the synthesis of quantitative outcomes assessing the effectiveness of treatments and practice. In contrast, a scoping review is an appropriate method to a) identify the scope of available literature on a given topic; b) provide an overview of concepts relating to the topic; and c) identify gaps in the literature. Given the limited literature exploring barriers and facilitators to e-health use by older adults, a scoping review of the available evidence, and evidence gaps, was considered most appropriate.

### Eligibility

Individual studies were included in the review if they: (i) were published in the English language; (ii) constituted outputs of empirical research (either quantitative, qualitative or mixed methods); (iii) were published in a peer-reviewed journal; and (iv) reported on participants aged 60 years and over. Studies were excluded if they: (i) were not written in the English language; (ii) constituted grey literature; (iii) were not published in a peer-reviewed journal; and (iv) reported on populations aged under 60 years. Sixty years was selected as the key age criterion, based on the United Nations definition of an “older” person, regardless of that person’s individual history or where in the world they live [32]. Articles which met the eligibility criteria were included regardless of journal rank and impact factor, to ensure identification of a wide range of methodologies; particularly qualitative methodologies, which remain underrepresented in high impact biomedical journals [33]. Studies were included if they made any form of reference to uptake, acceptance, attitudes, benefits, influences, perceptions, usefulness, determinants of use, experiences, expectations, and beliefs in relation to e-health use by older people. e-Health was defined as any electronic, mobile, online-delivered health or mental health service, including passive (e.g., health information webpage or patient portal) and active (e.g., clinician-moderated) therapy [18].

### Search strategy

A search of databases: CINAHL, Embase, Medline, Psychology and Behavioral Sciences, PsycINFO, and Scopus, was conducted by DBooth on 4th August 2020. No limit was placed on the date of databases searched. A combination of subject headings and keywords specific to each database was used in Medline, PsycINFO, Embase, and CINAHL. Keyword searches were used in Psychology and Behavior Science Collection and Scopus databases. See supplementary file for search strategy.

### Screening

**Fig. 1:**
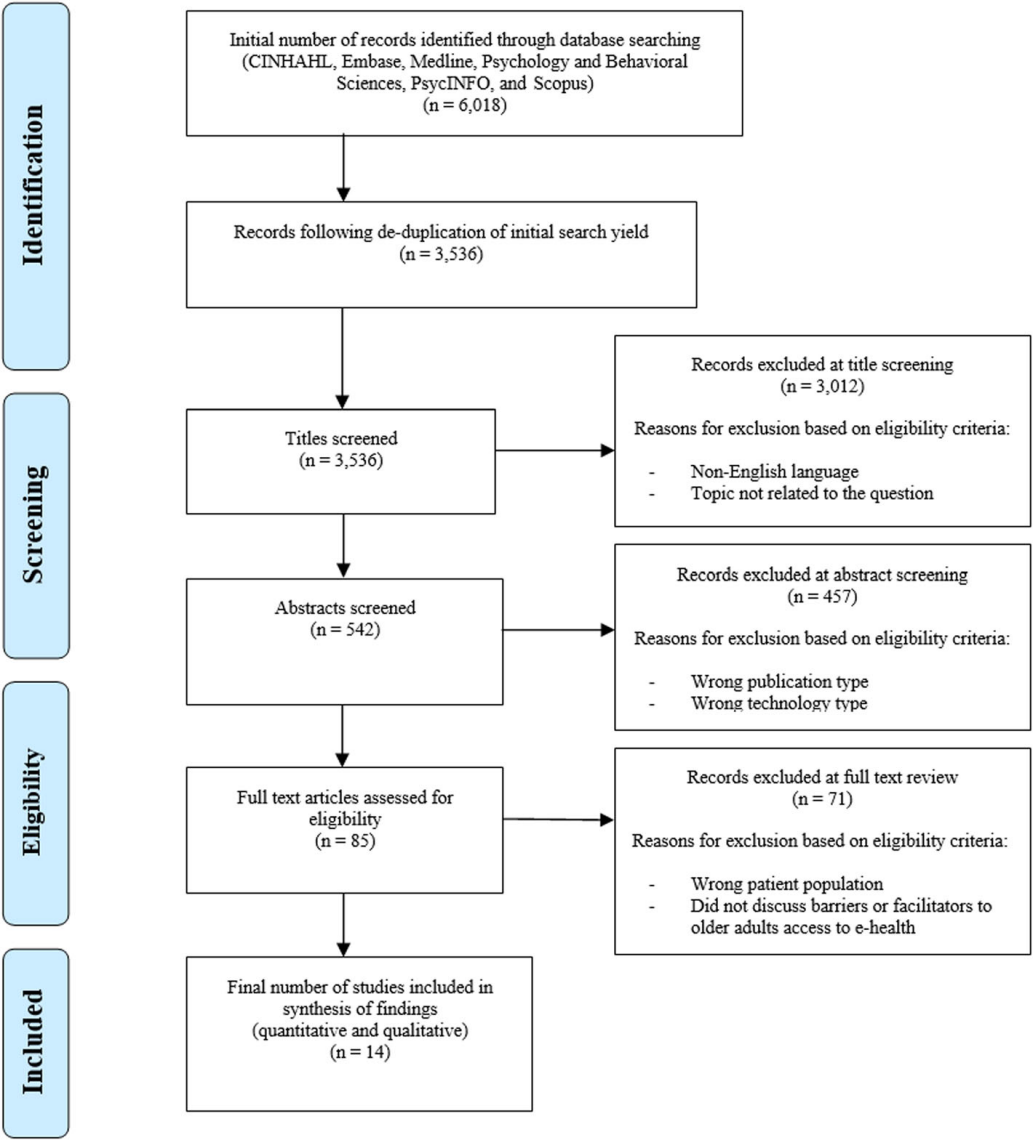
PRISMA flow chart of study selection

Figure 1 shows the PRISMA flow chart. A total of 3536 papers were identified and were uploaded to Covidence (https://www.covidence.org/), where all screening and data management was completed against the inclusion and exclusion criteria. After screening titles by the predefined eligibility criteria, 3012 were excluded, resulting in 542 papers. Following this, a further 457 papers were excluded based on abstract screening, leaving 85 papers for full text review, resulting in a total of 14 papers for extraction. It should be noted that the preliminary search for appropriate papers identified two studies with participants aged 50 years and older, which provided valuable information relating directly to the research question [34, 35]. A decision was made to include these studies, as the mean group age was greater than 60 years. JW performed the initial title and abstract screening phases of the review. Both JW and DBetts reviewed the full text publications for inclusions, with MH resolving any conflicts.

### Analysis

Data was extracted from the 14 included studies according to the following fields: author and year, field (e.g., chronic disease or mental health), study design, study focus e.g., (prevention of diabetes or depression intervention), description of population including important demographics such as rural location or physical disability, age range and mean, recruitment country, technology type (e.g., tablet or PC), service or intervention (e.g., pain management application), barriers to access, and facilitators to access. Tables 1 and 2 displays these data. After familiarisation with each of the papers, preliminary coding of three papers was completed by JW and DBetts, and a codebook was created to guide the analysis of the remaining 12 papers by JW. Following this, codes were cross-referenced and synthesised into five thematic categories by JW and DBetts, with consultation from MH to resolve discrepancies. Key themes were discussed with reference to the Unified Theory of Acceptance and Technology Use 2, briefly outlined below.

**Table 1:** Characteristics of included papers (*n* = 14)

Author & Year	Study design	Focus	Population (n, mean age)	Recruitment country	Technology
Bhattarai et al. (2020)	Qualitative interviews	App for pain self-management of arthritic pain	65+ years with arthritic pain (16, 73)	Australia	Smart phone
Bujnowska-Fedak & Pirogowicz (2014)	Quantitative survey	How to support elderly Polish people to access e-Health (preferences and attitudes)	60–90 years, supporters, and non-supporters of e-health (286, 74)	Poland	Any device with internet access
Cajita et al. (2018)	Qualitative interviews	Mobile health adoption in older adults with heart failure	66–83 years with heart failure (10, not reported)	USA	Smart phone
Coley et al. (2019)	Mixed methods	Prevention of cardiovascular disease and diabetes	65+ years with cardiovascular or diabetes risk factors (341, 68.7)	Finland, France & Netherlands	PC
Currie et al. (2015)	Qualitative interviews	Attitudes and acceptance of eHealth technologies by older rural people with chronic pain	60–74 years, rurally located people with chronic pain (4, not reported)	Scotland	PC, laptop, or tablet
de Veer et al. (2015)	Quantitative survey	Intention to use e-health	60–77 years (1014, not reported)	Netherlands	PC
Mishuris et al. (2014)	Qualitative interviews	Barriers to patient portal access - veteran specific	50–100 years, veterans (17, 61)	USA	PC or laptop
Nymberg et al. (2019)	Qualitative interviews	Beliefs, attitudes, experiences, and expectations of IT interventions for the prevention and treatment of chronic diseases	65–80 years with at least one chronic disease (15, 73)	Sweden	Any devise with internet access
Park et al. (2020)	Qualitative interviews	Perceptions and experiences of IT medication adherence	Range unknown, Veterans and non-veterans with history of coronary heart disease (28, 67)	USA	Smart phone
Pywell et al. (2020)	Qualitative interviews	Barriers to older adults’ uptake of mobile-based mental health interventions	53–77 years with low mood (10, 68)	England	Smart phone
Rasche et al. (2018)	Mixed methods	Barriers to using health apps	61–82 years, general population (95, 67)	Germany	Smart phones and tablet
Still et al. (2018)	Qualitative interviews	Experience of African-Americans’ using an app to manage hypertension	62–91 years with hypertension (21, 72)	USA	Smart phone
Van Middelaar et al. (2018)	Qualitative interviews	HATICE (Healthy ageing through internet counselling)	65–84 years with increased risk of cardiovascular disease (20, 71)	Netherlands	PC
Zibrik et al. (2015)	Mixed methods	Immigrant Chinese & Punjabi seniors’ barriers and facilitators to e-health	60–79 years. Punjabi, and Chinese immigrants (55, not reported)	Canada	PC

*Note.* PC = personal computer

**Table 2:** Overview of findings

Factor	Category	Barrier	Facilitator
Individual	Intrinsic	Ageing limitations: reduction of hearing, sight, memory, and fine motor control [34, 36–39]. Perceived self-efficacy [35, 38, 40, 41]. Lacking confidence in e-health [42]. Fear and dislike of technology [37]. No interest in learning [37, 42].	Desire to learn [34, 36–38, 41]. Motivation to make a lifestyle change [19, 43]. Altruism: wanting to contribute to scientific progress [19, 41, 43].
	Extrinsic	Lack of experience/skills with e-health [35, 37] or technology [36, 38, 41]. Lack of knowledge of e-health [34, 35]. Previous negative experience [40]. Unmet expectations [37]. Lack of need to change [34, 36, 37, 39]. Fear that traditional services my perish [37]. Disbelief in efficacy of e-health [37, 39, 42]. Lack of external accountability [44, 45]. Inability to incorporate into routine [44]. Required effort [35, 38]. Cultural limitations such as language barriers and e-health detracting from time with family [38].	Belief that e-health services are of benefit [19, 34, 37, 40]. Convenience of e-health [45]. Ability to incorporate into current routine [40, 44, 46]. Previous experience and skills [19, 35, 36, 40]. Previous experience with e-health and required skills [19, 35, 36, 40]. Positive experience with technology generally [37]. Opportunity to learn new information [43].
Technological	Functional	Small screen and text [44]. Small icons, lack of colour contrast [36]. Complex functionality [42]. Poor functionality [35, 37, 41].	Ease of use such as audio feedback, and large and clear visual display [35, 36, 40, 41].
	Content	Lack of alerts [41]. Alert fatigue: reminders/emails/texts [46]. Condescending and impersonalized communication, inability to respond to reminders [46]. Overwhelming and difficult to understand content [35, 38]. Too much content on one page [44]	Personalized content [37, 44–46]. Use of reminders/alerts [41, 44, 46]. Use of images [46].
	Availability	Lack of access to electronic equipment [38] Cost of electronic equipment and internet service [34, 36].	Free or low-cost electronic equipment [36].
Relational	Technological Support	No training/support to learn [36, 38]. No one to help troubleshoot issues [41]. Reliance on family for guidance, and lack of family’s patience and understanding while learning [38].	Training/support to learn [36–39, 41]. Dedicated coach for training and continued support [41]. Peer-to-peer platform to share experiences [44]. Option for family/carer to provide support [34].
	Social Support	Lack of social interaction [37, 45]. Absence of interpersonal communication [35]. Communication through technology considered an ‘inauthentic experience’ [35].	Socially inclusive and community-based information [38].
Environmental	Location	Poor/unreliable internet [45].	Availability to rural/remote populations [45].
Organizational	Privacy	Health information concerns [35, 42, 46].	
	Trust	Unknown accuracy of information [37, 38, 42]. Not knowing who people are communicating with [35]. Concern over management of emergency situations [37]. Concern over Western Medicine’s prioritization of medication [38].	Recommandation from physician [36, 43]. Content designed by experts in the field [45]. Access to specialists through platform [34]. Authenticity: platform with clear credentials [35].
	Data sharing	Lack of communication between health platforms [37].	Sharing of health information between health care providers [39, 44, 46].

*Note.* Individual = persons’ individual attributes including physicality, cognition, experience, skills, and knowledge; technological = the use of the technology, including device functionality, content, and availability; relational = person-to-person engagement and support; environmental = location context and characteristics; organizational = structure, capabilities, and development of the service

### Theoretical framework

The Unified Theory of Acceptance and Technology Use (UTAUT) is one of the most comprehensive and widely used technology acceptance models [47]. UTAUT proposes that behavioural intention to use technology is affected by an individual’s effort expectancy (degree to which the technology is perceived to be easy to use), performance expectancy (degree to which the technology is perceived to be useful), social influence (degree to which using the technology is supported by an individual’s social network), facilitating conditions (the degree to which an individual believes to have the resources to use the technology) [48]. UTAUT2 adds three additional constructs to the original UTAUT—hedonic motivation (degree to which the technology is perceived to be enjoyable), price value (degree to which the technology is perceived to be affordable and cost-effective) and habit (the degree to which technology use is influenced by the passage of time) [49]. UTAUT and UTAUT2 are most commonly applied using quantitative approaches. However, in this review UTAUT2 was applied as an analytical framework to facilitate deeper insights into the key findings from this review and identify areas for further research.

## Results

Of the 14 papers identified, 12 reported on barriers, and 13 reported on facilitators of e-health use in older adults. The characteristics of these papers are summarized in Table 1.

The barriers and facilitators to older adults accessing e-health were each mapped into five thematic categories (1) *individual*, including intrinsic and extrinsic; (2) *technological*, including functionality, content, and availability; (3) *relational*, including technological support and social support; (4) *environmental*, including location; and (5) *organizational*, including privacy, trust, and the sharing of data (see Table 2).

### Individual (*n* = 14)

#### Intrinsic

Intrinsic barriers (including physical, sensory, intellectual ability, and motivation) were discussed by nine of the included studies. Physical ageing was the most prevalent barrier to accessing e-health, with hearing and sight limitations being the most common [34, 36–38]. Concerns about memory were also reported [38], particularly with remembering passwords, and the acquisition of new information [39]. Additionally, the reduction of fine motor control (i.e., trembling hands) made it difficult to interact with devices, particularly those with small screens [34, 37]. Perceived self-efficacy regarding the use of technology was discussed as a barrier by four of the included studies. Discussion about perceived efficacy focused on: i) the difficulties of using technology [38] and e-health [40]; ii) concerns about the use of digital mental health technologies [35]; and iii) feelings of incompetence [41]. Other intrinsic barriers included a lack of interest in learning, and a fear or dislike of technology [37, 42].

Intrinsic facilitators were discussed by seven studies. Of these, five highlighted a willingness and desire to learn [34, 36–38, 41], finding that participants who articulated an innate sense of curiosity and interest in technology were more willing to use e-health, and more likely to engage and explore various e-health platforms. Other facilitators were a motivation and desire to make a lifestyle change [19, 43] and a desire to contribute to scientific progress by trialling e-health programs in the context of research [19, 41, 43].

#### Extrinsic

Extrinsic barriers (external factors outside the individual) were discussed by nine studies. These included inexperience with e-health [35, 37] or with computers/technology in general [36, 38, 41], and an overall lack of awareness of e-health opportunities [34, 35]. Some studies reported that participants had previous negative experiences [40] or unmet expectations [37] in relation to e-health services; a preference for traditional health care services [34, 36, 37, 39]; or a genuine fear that, if unused, traditional health services may cease to exist [37]. Stigma around e-health services in some studies extended to a disbelief in the reported advantages of technology [37], lack of confidence in the use of technology as a health service [42], and a belief that telephones (smart phones) are for telephone communication only and not for health services [39]. Other studies reported that the perceived lack of routine and structure (external accountability) provided by e-health services [44, 45] created a barrier to incorporate e-health into daily routines [44], and a perception that learning to engage with e-health involves more effort than reward [35, 38]. Cultural barriers, including second language difficulties and the cultural value of technologies detracting from time with family were also noted [38].

Extrinsic facilitators were identified by eleven studies. These included a perception that e-health services are of benefit [19, 34, 37, 40] and have the potential to support health care management [37], independent living [40], and self-managed care [39, 43, 46]. One study identified the convenience afforded by e-health programs, allowing participants to progress their care at their own pace and accommodating issues such as reduced mobility [45]. Three studies found that the ability to incorporate e-health into participant routines facilitated their use of these services [40, 44, 46].

Six studies focused on participants’ previous experiences of, and skills relating to, e-health programs [19, 35, 36, 40], finding that prior exposure to, or experience of, e-health [35] and previous positive experiences with technology more generally [37], facilitated the use of e-health in the future. A related finding was that for some participants the opportunity to learn new information acted as a facilitator for engaging with e-health [43].

### Technological (*n* = 11)

#### Functional

Six studies discussed functional barriers related to the design of e-health programs and their interface with older end users. Problematic features included small screen and text [44]; small icons and lack of colour contrast between text and background [36]; and complex functionality that assumes the user has experience with the technology [42]. Poorly functioning platforms, including problems with logging in and navigation [41], and faulty IT systems that did not function as intended [35, 37] were also barriers to use.

Functionality (ease of use) was identified as a facilitator to e-health use in four studies [35, 36, 40, 41]. For example, de Veer et al. highlighted the importance of platforms that are ‘pleasant’ to interact with, and Cajita et al. identified useful features for older adults, such as a large visual display and audio feedback for users [36, 40].

#### Content

Five studies discussed barriers relating to content, such as built-in reminder systems to reinforce e-health use. Lack of alerts or reminders was a barrier reported by van Middelaar et al. [41]. On the other hand, participants trialling a medication adherence application reported ‘alert fatigue’, from too many reminders [46]. Participants in this study also reported condescending communication (praise for taking medication), impersonal messages, and an inability to respond to messages (facilitates memory) as barriers to continued use [46]. Regarding service content, the large amount of information offered across e-health services was perceived as overwhelming and difficult to understand [35], particularly when the information included complex medical terminology [38]. Additionally, having too much content on one page was a barrier to use [44].

Five studies discussed the content of e-health services as facilitators, highlighting the need for specifically curated, personalized content, that aligns closely with user needs [37, 44–46]. Additionally, three studies found that e-health use was facilitated by reminders and alerts about content [41, 44, 46], and the use of images to facilitate memory and attention in relation to medication [46].

#### Availability

Barriers relating to e-health availability were discussed by three studies. These included a lack of access to the required electronic equipment (i.e., smart phone, tablet, or computer) [38] and the cost to purchase and upgrade this equipment, as well as the cost of an internet/mobile data or wi-fi service [34]. In particular, cost was a barrier for older adults who were on a limited or fixed income such as a pension [36]. Participants in Cajita et al. stated that the cost of the required equipment outweighed the perceived benefit of engaging with e-health [36]. In contrast, one study found that free, or low-cost, electronic equipment such as a computer or smart phone facilitated the use of e-health by older adults [36].

### Relational (*n* = 7)

#### Technological support

Three studies found that a lack of technological support (e.g., training, troubleshooting, and guidance) provided alongside e-health programs was a barrier to uptake. For example, participants in two studies stated that they would have felt more encouraged to use e-health if they were given adequate training and support in using the technology [36, 38]. Participants in another study were discouraged from using an online counseling platform because there was no support to troubleshoot issues [41]. Reliance on family for support and guidance, and a lack of patience and understanding from family members while participants were learning to use the mobile technology, was also highlighted as a barrier [38].

Seven studies identified technological support as a facilitator to e-health use by older adults. Five studies found that uptake was facilitated by training and support in relation to the technical aspects of a program [36–39, 41]. Findings highlighted the need for a dedicated coach to provide training, and continued feedback, to support participant engagement and progress through the e-health program [41]. Additionally, Bhattarai et al. found that peer-to-peer based platforms allowed participants to share knowledge and experience, thereby facilitating e-health engagement [44]; while Mishuris et al. found that family and carer support could facilitate e-health use [34].

#### Social support

Lack of social interaction was discussed in three studies as a barrier to e-health use. Not seeing a person face-to-face, whether it be a doctor or peers in a group setting, was a key deterrent to e-health uptake [37, 45]. For participants using a mobile-based mental health intervention, the lack of interpersonal communication was perceived to detract from the therapeutic process, with communication via technology considered an ‘inauthentic’ experience for this age group [35].

One study found that inclusive, community-based approaches to designing and implementing e-health supported uptake by participants, such as peer-led health information sessions, and receiving information from the community was particularly important for diverse ethnocultural groups [38].

### Environmental (*n* = 1)

#### Location

Unreliable or unavailable internet services in rural and remote locations, were discussed as barriers in one study [45]. On the other hand, one study focusing on older adults in rural and remote communities [45] addressed environmental factors relating to location, finding that e-health reduced the need to travel long-distances to health care appointments.

### Organisational (*n* = 10)

#### Privacy

Concerns about privacy and security were raised by participants in three studies [35, 42, 46]. In one study, 28% of respondents surveyed viewed privacy as a barrier to using e-health [42]. Additionally, participants using a mental health intervention expressed concerns about who was accessing their health information, and how information was being shared with practitioners [35]. No studies identified specific facilitators relating to privacy.

#### Trust

Mistrust of e-health was reported across four studies, with a lack of trust in the accuracy of the information contained in e-health being the greatest concern [37, 38, 42]. Other issues of trust related to participants’ uncertainty about who they were communicating with, particularly about mental health issues [35]; and appropriate management of emergency situations [37]. Additionally, Chinese and Punjabi immigrants in Zibrik et al.’s study expressed a distrust in e-health due to a perceived association with Western medicine’s prioritization of medication over natural therapies [38].

Five studies discussed trust, with two identifying that e-health services recommended by a physician were more likely to be used by older adults [36, 43]. In one study, this recommendation took the form of a letter inviting patients to participate in an e-health program from their trusted practitioner [43]. Further, participants were more likely to trust e-health services that were designed by experts in the field [45], provided access to specialists [34], and provided a clear purpose and transparent credentials [35].

#### Data sharing

One paper identified a lack of information communication between health platforms and professionals as a barrier, with participants expressing a desire for e-health services to be streamlined, and information to be shared [37]. Supporting this finding, [37] three studies in which e-health platforms had the capability to share data with health services found that this facilitated the use of e-health [42].

## Discussion

This scoping review sought to explore barriers and facilitators to the use of e-health by older adults, with the aim of informing future development and uptake of digital health and mental health interventions for this age group. The Unified Theory of Acceptance and Use of Technology2 (UTAUT2) was used as an analytical framework to further examine the findings and identify opportunities for future research.

Analysis of the five thematic categories resulted in three broad implications for the development of future e-health services for older adults. These relate to the 1) design of the e-health service; 2) training and education provided to increase e-health literacy; and 3) perceived authenticity of the service. Contextual implications are discussed as a sub-theme.

### Design of the e-health service

Consideration of the specific needs of older adults in the design of digital health services was one of the most significant factors impacting uptake and ongoing use of e-health services in this review. Consistent barriers related to the functionality of e-health platforms and problems with the user interface, such as small screens, text, and images. These barriers reflect a lack of consideration of physical difficulties associated with ageing, such as poor eyesight, hearing, and memory, which can hinder older people’s engagement. Findings also showed that older people can become overwhelmed by new information and alerts, and by challenges associated with altering or customising the user interface to their individual needs, creating barriers to uptake. Conversely, when the design of e-health services addresses the needs of older adults, engagement increases. Specifically, e-health services that were accessible, pleasant to use, had larger screens, such as a tablet or desktop/laptop, larger font size, audio features, notifications, and diverse, curated content showed greater uptake. Based on these findings, the following features should be considered in the design of e-health services: i) offering services that are accessible across multiple technologies including tablets and computers; ii) features such was audio feedback, large text size, and a notification system that allows users to set how and when they are notified, enabling engagement with platforms in a manner that best suits the individual; and iii) including wide and diverse information that can be curated for the user based on their circumstances, reducing the need for navigation through content that may be irrelevant and overwhelming, while still offering a platform that addresses multiple health needs without requiring users to engage with different platforms, services, or professionals.

Findings from this review suggest that both useability and usefulness are important factors to consider when designing future e-health services. These factors align with the constructs of individual effort expectancy and performance expectancy in the UTAUT2 framework. In fact, one study included in this review applied the UTAUT, finding that effort expectancy and performance expectancy were both highly related to older people’s intention to use e-health [40]. It should be noted that findings from other studies differ, suggesting that for older people, effort expectancy is more important than performance expectancy in predicting the uptake of digital technologies [50], however, this study did not specifically focus on the use of e-health services.

Useability and usefulness have been recognized as important components of successful e-health uptake in the wider literature [51], with De Rouck et al. [52] noting that a thorough understanding of the factors that impact on the useability and usefulness of e-health services for specific end users would support technological design and effectiveness. Since older adults are not a homogenous group [2], their physical needs and ability to engage with digital platforms can vary. Consideration of age-related factors and allowing older adults to customize platform interfaces would provide them with more options to engage. To address these issues, findings from this review suggest that future e-health developers should not only consider the design elements described earlier in this discussion but should actively incorporate the feedback of older adults in their design, engagement, and delivery strategies. This process of consultation can be achieved using focus groups, individual interviews or surveys, and pilot studies – all of which can occur both pre- and post-development of e-health platforms.

### Training and education to increase e-health literacy

Alongside design, a significant factor influencing the successful uptake of e-health by older adults was training and education in how best to use the technology to their advantage. The ability to use and benefit from e-health, known as e-health literacy, is an important part of ensuring the effectiveness of e-health program engagement and outcomes across the lifespan [53, 54]. In this review, effective training, and education to develop e-health literacy took two distinct forms—providing practical skills to support older adults’ use of e-health programs and addressing misconceptions or previous negative experiences with e-health programs.

In relation to practical skills, common barriers were a lack of i) previous experience, ii) training on how to use the technological features of the program, and iii) access to formal or informal supports to troubleshoot problems. Yet, older adults who were provided with support, guidance, and training were more likely to express positive associations with e-health. Specific examples of successful training and support included; addressing issues of discouragement and inexperience by providing a dedicated coach for initial and ongoing guidance; helping to build trust, encouragement, and motivation [41]; addressing a lack of basic computer skills by facilitating and offering low-cost group computer classes [38]; and including family and carers in initial training sessions so they could provide informal ongoing support [34]. Additionally, where support and training focused on the potential benefits of e-health, older adults were less likely to perceive it as difficult, incompatible with their current health and lifestyle needs, or ineffective as a treatment platform.

Application of the UTAUT2 suggests that providing older people with training and education in the use of e-health technologies may facilitate effort expectancy, performance expectancy and facilitating conditions. They also suggest that social influence may play a role in supporting ongoing engagement with e-health, supporting findings from a previous systematic review [55]. In contrast, de Veer et al. [40] found that social influence had no impact on e-health uptake, after beliefs about performance expectancy and effort expectancy had been taken into account. According to Venkatesh et al. [48] social influence only plays a role in a mandatory context. However, findings from this review suggest that receiving information and support from community members was important for older people, particularly those from diverse ethnocultural groups [38]. Future research should therefore explore the impact of social influence on e-health uptake by older people from specific cultural groups.

### Authenticity

Findings from this review suggested that e-health uptake is enhanced when e-health services and service providers are perceived to be authentic (trustworthy and credible). Older adults were less likely to engage with e-health services when they were concerned about how their privacy would be protected. Additionally, some older adults expressed uncertainty about the appropriate sharing of their personal information with other health services, with one study reporting that participants favoured e-health programs that were streamlined across traditional service settings and shared pertinent information with appropriate health professionals across these settings [37]. The importance of establishing trust in e-health has been increasingly recognised as a key challenge for the field, with previous research suggesting that consumer confidence in information security and privacy is likely to influence how they choose to engage [56]. These concerns could be addressed by employing strategies to strengthen the authenticity of the e-health program. Strategies could include referrals to e-health services from a trusted source such as a general practitioner or mental health service provider [36, 43], providing access to health and mental health specialists [34], and ensuring that e-health services practice effective collaboration in the management and sharing of relevant health information [39, 44, 46].

Findings from this review suggest that the impact of variables such as perceived credibility and trustworthiness on e-health uptake by older adults may warrant further exploration. While the UTAUT2 does not include a specific construct relating to trust, a recent study by [27] extended the UTAUT2 by adding two important factors, mass media (channels of communication—whether written, broadcast, or spoken—that reach a large audience) and trust (the subjective expectation with which consumers believe that a specific transaction occurs in a way consistent with their expectations). Application of these constructs in a small sample of Jordanian community members (*n* = 7) found that the adoption of a mobile banking technology was positively and significantly influenced by the mass media (television, radio and internet promotion) and trust (security and privacy of the mobile banking service) [27]. These additional constructs shed new light on the findings of this review. Viewed in combination, is possible to infer that targeted public health media campaigns to raise the profile, relevance, and credibility of e-health services, and articulate how to evaluate the credibility and utility of these services, may be effective in addressing some of the barriers to e-health uptake by older adults.

### Contextual considerations

So far, this discussion has focused on three broad implications for the development of e-health services for older adults. While not as prominent in the literature, the sub-theme of contextual considerations nonetheless offered important insights for future development of e-health programs for older adults.

In some studies, financial factors were highlighted as a barrier to accessing e-health programs. Older adults who were retired, on a fixed income, or who lived in a remote location were less likely to engage with e-health programs. The ability to use technology was also restricted by the type of access to the internet, the cost of owning or upgrading a computer, or a perception that the cost of accessing e-health programs outweighed the benefits. Analysis of these findings using the UTAUT2 suggest that price value may be an important facilitating condition that plays a role in the uptake of e-health technologies by older people. Further research applying the UTAUT2 with this population is needed to determine the predictive power of this construct.

Findings from this review highlight the important issue of equity in accessing e-health, where a possible digital divide exists beyond age or generational issues [18]. The notion of a digital divide broadly refers to the separation that can exist between those who have access to, and the ability to understand diverse technological resources, and those who do not [57]. Research in this area has found that structural inequalities such as low socioeconomic status, ethnicity, and education levels, often contribute to such disparities in the use of e-health programs [58, 59]. Further, Beard [12] suggested that challenges of appropriate resourcing and access to technology are likely to be more significant for older adults than for other groups; an observation supported by this review. Conversely, e-health also holds great potential for enhancing access to health and mental health programs for older adults, particularly those with disabilities or those who live in remote locations, with limited transport options.

### Gaps in the literature and opportunities for future research

While literature on the impacts and efficacy of e-health for older adults is growing [19, 20], to date, few studies have focused on understanding the practical and conceptual barriers and facilitators for older adults in accessing e-health services. Given the rapid increase in population ageing, and the complex health and mental health challenges older people can experience, future research exploring the potential for e-health to respond to these challenges is essential. Research with a focus on digital *mental* health interventions for older people is needed, as this review identified only one study that focused on the use of e-mental health by older people. This finding is concerning given the prevalence of mental health concerns in older populations [4, 5], the increased risk of physical health problems in older adults with mental health problems [60], and Australian data indicating that older adults are the least likely of all age groups to access mental health services [61]. Future research is also needed to explore broader environmental and contextual factors impacting on e-health use by older people, as the existing literature tended to focus on individual, relational and design-related factors. Findings from one study suggested that including older people in the process of designing and developing e-health services may enhance their relevance for, and use by, this population. More research is needed to explore how older adults can best be included in the e-health design process.

Additional gaps in the literature were highlighted when applying the UTAUT2. Notably, findings from this review did not find evidence that specifically supported the constructs of habit and hedonic motivation. While three studies did find that e-health uptake was enhanced when participants were able to integrate the e-health service into their pre-existing routines, this finding does not directly address the construct of habit (the length of time from initially adopting and using e-health). Further research could address this gap by exploring whether the passage of time has an impact on e-health engagement by older people. While e-health services are not conventionally designed to be enjoyable, future research could also investigate what aspects of hedonic motivation might support engagement with these services. Finally, findings from this review suggested that the constructs of price value and social influence may facilitate the uptake of e-health services by older people. Of particular importance was the finding that these constructs may impact on specific groups of older people who are already experiencing higher levels of disadvantage, such as older people on low or fixed incomes, or older people from cultural or ethnic minority groups. This highlights an urgent need for future research examining factors that facilitate or hinder the use of e-health services by specific groups of older people, who may be particularly vulnerable or marginalised. Combining UTAUT2 with normative theories of social justice and equity may facilitate such efforts [47].

### Limitations

This review has several limitations. Firstly, as non-English publications were excluded, any pertinent non-English language publications are likely to have been missed, possibly resulting in a culturally biased review. Secondly, while the inclusion criteria for this review enabled identification of a wide range of literature the use of broader search terms means that studies focused on more specific, narrow subject areas may have been missed. Finally, while PRISMA-ScR guidelines were adhered to at every stage of this review, the protocol was not registered.

## Conclusion

Consideration of the specific barriers and facilitators that influence the use of e-health by older adults is critical to improve their use of e-health programs, and to realise the potential of technology to ameliorate the challenges associated with traditional healthcare for this group. Findings from this review suggested that older adults are more likely to use e-health services that are cognizant of their physical and functional needs, provide appropriate education and training to engage with e-health, address previous negative experiences of, and misconceptions about, digital health technologies; and employ strategies to enhance the perceived trustworthiness and credibility of e-health. Further research is needed to explore the practical and conceptual barriers and facilitators for older adults in accessing e-health.

## Supplementary Information

**Additional file 1.**

## Data Availability

All data generated or analysed during this study are included in this published article [and its supplementary information files], including PRISMA checklist, and raw extraction file.
