# Supplementary material for: Barriers and facilitators to the use of e-health by older adults: a scoping review
Source: BMC Public Health. 2021 Aug 17;21:1556. doi: 10.1186/s12889-021-11623-w (PMC8369710; doi:10.1186/s12889-021-11623-w)
Supplement: Supplementary file 1 — Additional file 1. [file 12889_2021_11623_MOESM1_ESM.docx]

Supplementary File: search strategy

Database(s): **Ovid MEDLINE(R) and Epub Ahead of Print, In-Process & Other Non-Indexed Citations and Daily**1946 to August 04, 2020
Search Strategy:

| **#** | **Searches** | **Results** |
| --- | --- | --- |
| 1 | "Aged, 80 and over"/ or Aged/ | 3121915 |
| 2 | Geriatrics/ | 29998 |
| 3 | ((older or elderly or senior) adj5 (adult* or Australian* or men or women or male* or female* or man or woman or person* or people or population)).mp. | 265308 |
| 4 | (geriatric* or pensioner* or "over 60*" or "old* age*" or "over sixt*").mp. | 195724 |
| 5 | 1 or 2 or 3 or 4 | 3283332 |
| 6 | Mobile Applications/ | 6001 |
| 7 | (mobile intervention* or mobile based).mp. | 625 |
| 8 | Internet/ | 72955 |
| 9 | exp Telemedicine/ | 29065 |
| 10 | Information Technology/ | 379 |
| 11 | (internet or web based or communication technolog* or "e health" or ehealth or mhealth or "m health" or “e mental health” or "online support" or "e communit*" or "health app*" or "mobile app*" or "interactive program*" or "online peer" or "intervention app*").mp. | 149156 |
| 12 | "digital technolog*".mp. | 1976 |
| 13 | 6 or 7 or 8 or 9 or 10 or 11 or 12 | 170638 |
| 14 | (intervention* or program* or therap* or treatment* or studies or study).mp. | 16724596 |
| 15 | ((recruit* or participa* or respond*) and (barrier* or uptake* or confidence)).mp. | 205939 |
| **16** | **5 and 13 and 14 and 15** | **1489** |

Database(s): **APA PsycInfo**1806 to July Week 4 2020
Search Strategy:

| **#** | **Searches** | **Results** |
| --- | --- | --- |
| 1 | ((older or elderly or senior) adj5 (adult* or Australian* or men or women or male* or female* or man or woman or person* or people or population)).mp. | 109155 |
| 2 | geriatric*.mp. | 45526 |
| 3 | pensioner*.mp. | 245 |
| 4 | over 60*.mp. | 2808 |
| 5 | over sixt*.mp. | 182 |
| 6 | exp Geriatrics/ or old* age*.mp. | 37495 |
| 7 | 1 or 2 or 3 or 4 or 5 or 6 | 150421 |
| 8 | Mobile Applications/ | 879 |
| 9 | Computer Mediated Communication/ | 5864 |
| 10 | (mobile intervention* or mobile based).mp. | 284 |
| 11 | internet/ | 29026 |
| 12 | (internet or web based or communication technolog* or e health or ehealth or mhealth or m health or e mental health or online support or e communit* or health app* or mobile app* or interactive program* or online peer or intervention app*).mp. | 85513 |
| 13 | exp telemedicine/ | 8792 |
| 14 | "information and communication technology"/ or digital technology/ | 9523 |
| 15 | 8 or 9 or 10 or 11 or 12 or 13 or 14 | 93505 |
| 16 | (intervention* or program* or therap* or treatment* or studies or study).mp. | 2856935 |
| 17 | exp Experimental Subjects/ | 4266 |
| 18 | ((recruit* or participa* or respond*) and (barrier* or uptake* or confidence)).mp. | 945464 |
| 19 | 17 or 18 | 161983 |
| **21** | **7 and 15 and 16 and 19** | **1374** |

Database(s): **Embase**1947 to present
Search Strategy:

| **#** | **Searches** | **Results** |
| --- | --- | --- |
| 1 | aged/ or very elderly/ | 3173708 |
| 2 | geriatrics/ | 38352 |
| 3 | ((older or elderly or senior) adj5 (adult* or Australian* or men or women or male* or female* or man or woman or person* or people or population)).mp. | 360864 |
| 4 | (geriatric* or pensioner* or "over 60*" or "old* age*" or "over sixt*").mp. | 296510 |
| 5 | 1 or 2 or 3 or 4 | 3422953 |
| 6 | mobile application/ or mobile health application/ | 12130 |
| 7 | (mobile intervention* or mobile based).mp. | 726 |
| 8 | Internet/ | 109169 |
| 9 | exp telemedicine/ or telehealth/ | 47237 |
| 10 | information technology/ | 10771 |
| 11 | (internet or web based or communication technolog* or e health or ehealth or mhealth or m health or e mental health or online support or e communit* or health app* or mobile app* or interactive program* or online peer or intervention app*).mp. | 199252 |
| 12 | "digital technolog*".mp. | 2438 |
| 13 | 6 or 7 or 8 or 9 or 10 or 11 or 12 | 243947 |
| 14 | (intervention* or program* or therap* or treatment* or studies or study).mp. | 24984671 |
| 15 | ((recruit* or participa* or respond*) and (barrier* or uptake* or confidence)).mp. | 269193 |
| **16** | **5 and 13 and 14 and 15** | **1821** |

**CINAHL**

| **#** | **Query** | **Results** |
| --- | --- | --- |
| S1 | (MH "Aged") OR (MH "Aged, 80 and Over") | 828,777 |
| S2 | (MH "Geriatrics") | 6,211 |
| S3 | ((older or elderly or senior) n5 (adult* or Australian* or men or women or male* or female* or man or woman or person* or people or population)) | 238,514 |
| S4 | (geriatric* or pensioner* or "over 60*" or "old* age*" or "over sixt*") | 159,095 |
| S5 | S1 OR S2 OR S3 OR S4 | 928,784 |
| S6 | (MH "World Wide Web Applications") OR (MH "Mobile Applications") | 13,009 |
| S7 | (MH "Internet") | 49,201 |
| S8 | (MH "Telehealth+") | 23,868 |
| S9 | (MH "Information Technology") | 13,924 |
| S10 | (internet or web based or communication technolog* or "e health" or ehealth or mhealth or "m health" or “e mental health” or "online support" or "e communit*" or "health app*" or "mobile app*" or "interactive program*" or "online peer" or "intervention app*") | 105,922 |
| S11 | "digital technolog*" | 1,209 |
| S12 | S6 OR S7 OR S8 OR S9 OR S10 OR S11 | 129,645 |
| S13 | (intervention* or program* or therap* or treatment* or studies or study) | 3,960,574 |
| S14 | ((recruit* or participa* or respond*) and (barrier* or uptake* or confidence)) | 132,275 |
| **S15** | **S5 AND S12 AND S13 AND S14** | **1,334** |

**Psychology and Behavioural Sciences Collection**

( (geriatric* or pensioner* or "over 60*" or "old* age*" or “over sixt*”) or ((older or elderly or senior) n5 (adult* or Australian* or men or women or male* or female* or man or woman or person* or people or population)) ) AND (intervention* or program* or therap* or treatment* or studies or study ) AND ( ((recruit* or participa* or respond* or barrier* or uptake* or confidence) ) AND ( "digital technolog*" or “web based” or “information technology” or telemedicine or telehealth or internet or “mobile app*” or “health app*” or “mobile intervention*” or “mobile based” or “communication technolog*” or “web based” "e health" or ehealth or mhealth or "m health" or “e mental health” or "online support" or "e communit*" or "health app*" or "mobile app*" or "interactive program*" or "online peer" or "intervention app*")

**SCOPUS**

TITLE-ABS-KEY ( geriatric* OR pensioner* OR "over 60*" OR "old* age*" OR "over sixt*" OR ( ( older OR elderly OR senior ) W/5 ( adult* OR australian* OR men OR women OR male* OR female* OR man OR woman OR person* OR people OR population ) ) ) AND TITLE-ABS-KEY ( intervention* OR program* OR therap* OR treatment* OR studies OR study ) AND TITLE-ABS-KEY ( recruit* OR participa* OR respond* OR barrier* OR uptake* OR confidence ) AND TITLE-ABS-KEY ( ( "digital technolog*" OR "web based" OR "information technology" OR telemedicine OR telehealth OR internet OR "mobile app*" OR "health app*" OR "mobile intervention*" OR "mobile based" OR "communication technolog*" OR "web based" OR "information technology" OR telemedicine OR telehealth OR internet OR "mobile app*" OR "health app*" OR "mobile intervention*" OR "mobile based" OR "communication technolog*" OR "web based" "e health" OR ehealth OR mhealth OR "m health" OR "e mental health" OR "online support" OR "e communit*" OR "health app*" OR "mobile app*" OR "interactive program*" OR "online peer" OR "intervention app*" ) )
